# Understanding the Links Between Self-Report Emotional Intelligence and Suicide Risk: Does Psychological Distress Mediate This Relationship Across Time and Samples?

**DOI:** 10.3389/fpsyt.2018.00184

**Published:** 2018-05-08

**Authors:** Sergio Mérida-López, Natalio Extremera, Lourdes Rey

**Affiliations:** ^1^Department of Social Psychology, University of Málaga, Málaga, Spain; ^2^Department of Personality, Assessment and Psychological Treatment, University of Málaga, Málaga, Spain

**Keywords:** emotional intelligence, psychological distress, suicide risk, mediator model, prospective design

## Abstract

**Objective:** In the last decades, increasing attention has been paid to examining psychological resources that might contribute to our understanding of suicide risk. Although Emotional Intelligence (EI) is one dimension that has been linked with decreased suicidal ideation and behaviors, we detected several gaps in the literature in this area regarding the research designs and samples involved. In this research, we aimed to test a mediator model considering self-report EI, psychological distress and suicide risk across samples adopting both cross-sectional and prospective designs in two independent studies.

**Method:** In Study 1, our purpose was to examine the potential role of psychological distress as a mediator in the relationship between self-report EI and suicide risk in a community sample comprised of 438 adults (270 women; mean age: 33.21 years). In Study 2, we sought to examine the proposed mediator model considering a 2-month prospective design in a sample of college students (*n* = 330 in T1; *n* = 311 in T2; 264 women; mean age: 22.22 years).

**Results:** In Study 1, we found that psychological distress partially mediated the effect of self-report EI on suicide risk. More interestingly, findings from Study 2 showed that psychological distress fully mediated the relationship between self-report EI and suicide risk at Time 2.

**Conclusion:** These results point out the role of psychological distress as a mediator in the association between self-report EI and suicide risk. These findings suggest an underlying process by which self-report EI may act as a protective factor against suicidal ideation and behaviors. In line with the limitations of our work, plausible avenues for future research and interventions are discussed.

## Introduction

In the last decades, the literature on individual differences regarding health and well-being has been expanding rapidly, thereby leading to a large body of research on psychological resources associated with mental health outcomes [e.g., (1, 2)]. Emotional Intelligence (EI) is one dimension that has shown robust associations with health-related outcomes (3), thereby constituting a particularly relevant topic in psychiatric research (4).

Two main theoretical approaches are found to build the framework for this construct: trait EI and ability EI. These views play a major role in the assessment of EI together with its training [for a review, see e.g., 5)]. In fact, there is a growing consensus in distinguishing three main construct-method pairings considering the model of EI: self-report mixed EI tests, performance-based ability EI instruments and self-report ability EI tests (6, 7). On the one hand, models of trait EI define this construct as a personality trait regarding the person's tendency to manage his or her emotional states (8). Therefore, researchers following this approach tend to use self-report mixed EI instruments. On the other hand, ability EI is referred to as a set of abilities that allow people to effectively deal with emotions (9).

According to the ability model proposed by Mayer and Salovey (10), EI is defined as “the ability to perceive accurately, appraise, and express emotion; the ability to access and/or generate feelings when they facilitate thought; the ability to understand emotion and emotional knowledge; and the ability to regulate emotions to promote emotional and intellectual growth” (10). Thus, the ability EI approach suggests implications on EI training as emotional abilities might be more susceptible to being developed and learned (9). Following the ability model of EI, performance-based ability EI tests are often used together with instruments referred to as self-report ability EI tests. In line with previous studies (11), a widely used self-report ability EI measure (i.e., Wong and Law Emotional Intelligence Scale) was chosen because it is relatively short, reliable and easy to administer. Besides, this instrument provides unique access to emotional-affective processes given by self-report ability EI tests.

### EI and suicide risk

While suicide is considered as a public health concern because of its alarming prevalence, suicidal thoughts and behaviors represent significant indicators of suicide risk (12). In this sense, the phenomenon of suicide has been argued as a continuum [e.g., (13)]. In addition, two populations have received particular attention in psychiatric research regarding the leading prevalence of deaths caused by suicide in both populations (12, 13). On the one hand, community samples constituted of middle-aged adults are required to deal with psychosocial events (e.g., loss of job, marriage, or relationship breakdown or financial stress) that are linked to increased suicide risk in this age group (14, 15). On the other hand, college students constitute a population at high risk of suicidal thoughts and behaviors (16). As some authors have argued, the university context represents a key transitional period often perceived as a stressful time of change, thereby influencing students' suicidal thoughts and behaviors (13).

Because the perceived ability to deal with affective information has been highlighted as a relevant factor regarding health and well-being indicators [e.g., (7, 17)], it is not surprising that findings from several studies have reported significant associations between self-report ability EI and suicide risk. For instance, Abdollahi et al. (18) found that self-report ability EI buffered the association between perceived stress and suicidal ideation among depressed adolescent inpatients. In this context, Abdollahi and Talib (19) argued the protective role of self-report ability EI against suicidal ideation because of its negative associations with rumination processes. Similar findings have been found on the relationship between self-report ability EI and suicide risk indicators among college students (20, 21) and community samples (22). With respect to performance-based ability EI tests, similar results have been reported in a study with adolescents (23). More recently, Paradiso and colleagues used a well-known performance-based ability EI test (i.e., Mayer-Salovey-Caruso Emotional Intelligence Test; MSCEIT, Version 2.0) in a study with a clinic sample of veterans (24). Findings showed that suicidal thoughts were linked to lower emotion processing. As noted above, existing literature on EI and suicide has focused on the ability model of EI using both self-report ability EI tests and performance-based instruments. In sum, there is a growing body of research suggesting that the manner in which people deal with emotional information contributes to an explanation of suicide risk.

### Psychological distress as a potential mediator between EI and suicide risk

In identifying risk factors linked with suicidality, impaired mental health constitutes identifiable vulnerabilities that increase the likelihood of suicide [e.g., (12, 25)]. In this context, prior research has reported the predictive validity of psychological risk factors on suicide risk among college students [e.g., (16)] or community samples [e.g., (26)]. Finally, the deleterious impact over time of psychological symptomatology on suicidality has been reported (27).

A broad association between EI and psychological distress indicators suggests that the perceived ability to deal with emotions is linked to individuals' psychological adjustment and adaptation [e.g., (2, 3)]. In addition, EI has been found to be involved in psychological distress processes beyond the influence of personality traits (4, 28). According to the EI framework, emotionally intelligent individuals manage their emotions in a better way than those with lower EI (5). Consequently, people with higher EI tend to adopt more adaptive regulatory strategies that are, in turn, negatively associated with negative affect and psychological distress [e.g., (29)]. Conversely, emotion dysregulation is considered a factor contributing to affective vulnerabilities that are in the basis of suicide risk (12, 30) and nonsuicidal self-injury (31). This latter risk factor has shown robust associations with increased desire for, and capability of, suicide across samples (32).

Even though researchers have focused efforts on identifying the buffering role of emotional abilities in understanding the associations between psychological risk factors such as perceived stress or depression (18, 21) and increased suicide risk, no study has examined a mediator model beyond the direct associations between these variables. In other words, there is a need for research to delineate the mechanisms through which EI might act as a protective factor to reduce suicidal thoughts and behaviors (33).

### Purpose of the present research

As noted above, we found several gaps in the literature on EI and suicide risk that motivate our work. First, although EI is negatively related to suicide risk, the mechanisms by which EI relates to suicidal thoughts and behaviors remain unclear. Second, most of studies examining self-report EI and suicide risk indicators relied on adolescent and college student samples [e.g., (20, 21)]. Thus, studies examining the associations between EI and health indicators in more heterogeneous samples are needed to confirm the validity of these results (17, 34). Finally, current findings in the literature on EI and suicide risk share a limitation derived from the use of cross-sectional designs. In short, previous studies have failed to capture change over time and left the question of causal direction unanswered. Therefore, the findings from prospective studies may provide clarity on causal mechanisms between EI and suicide risk (22, 33). Besides, analyzing EI at Time 1 with reported suicidal ideation and behavior at Time 2 offers a more stringent test of the impact of EI on suicide risk. Moreover, this prospective design can reveal the existence of stable relationships that might not be detected when constructs are measured at only one point in time.

To begin to fill these gaps, the objective of the present work was threefold. First, we aimed to examine the associations between self-report EI, psychological distress and suicidal thoughts and behaviors in two different populations (community sample and college students) to confirm the generalizability of our results. Second, we aimed to examine whether psychological distress would mediate the relationship between self-report EI and suicide risk. Third, we analyzed the proposed mediator model considering both cross-sectional and prospective designs in two independent samples.

**Figure 1:**
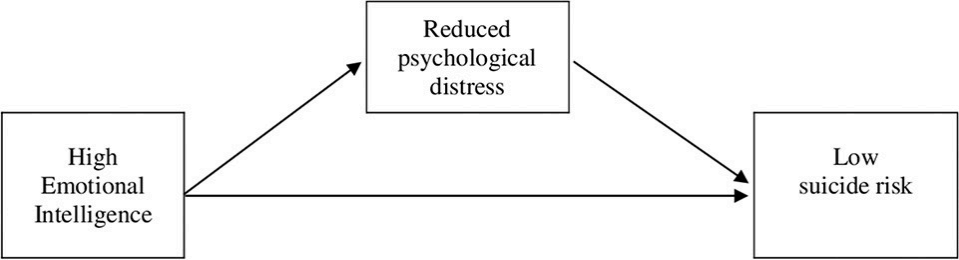
Proposed model of the role of psychological distress in explaining the relationship between emotional intelligence to suicide risk.

We undertook two studies aiming to achieve the above-described objectives. Based on prior research, we expected that: (a) direct associations would exist between self-report EI, psychological distress and suicide risk, and (b) psychological distress would operate as a mediator of the relationship between self-report EI and suicide risk. In Study 1, we explored this proposed mediator model in a community sample. In Study 2, we aimed to verify prospectively the proposed model in a sample of college students, that is, taking a temporal mediation approach to examine the effects of self-report EI on Time 2 suicide risk over a 2-month period. Our proposed mediator model is shown in Figure 1.

## Study 1: materials and methods

### Participants and procedure

A convenience sample of 438 Spanish speaking adults (270 women; 61.60%) living in southern Spain took part in this study. The ages of participants ranged from 17 to 62 years, with a mean of 33.21 years (*SD* = 11.68). The marital status of the participants was: 57.3% single, 27.9% married, 7.5% separated/divorced, 1.8% widow/widower and 4.8% coupled. Three subjects did not indicate their marital status.

Participants were recruited with the help of psychology students at University of Malaga. In this sense, respondents were invited to participate through a snowball sampling technique via the researchers' and undergraduates' personal and professional contacts. These students were given copies of the surveys and received instructions from the teaching staff regarding how to administer the questionnaire correctly. Overall, participants were aware that by completing the questionnaires they were providing informed consent to use this data in the present research. No financial compensation was offered to the subjects for their participation. Common inclusion criteria were being aged above 18 years old at the time of this survey and willingness to participate in the research. Exclusion criteria were illiteracy in Spanish and not being interested at participating in filling in the individual, confidential and voluntary questionnaire. Participants received oral and written information about the aims of the study and were fully informed about the anonymity and the voluntary nature of the research so that potential coercion was avoided. Most importantly, it was made clear that they could stop participating in case they got distressed filling in the questionnaire. Once the participants completed the questionnaires at home, the students returned them to the teaching staff for statistical processing. The questionnaires included written information on the main purpose of the study and standard instructions on how to complete the tests. Completing the surveys lasted 15 minutes on average. In addition to sociodemographic data (age, gender and marital status), the questionnaires included well-validated scales assessing the main study variables.

### Measures

To assess for self-report EI we used the Wong and Law Emotional Intelligence Scale [WLEIS; 35)]. This instrument assesses four dimensions: self-emotion appraisal, other-emotion appraisal, using of emotion, and regulation of emotion (e.g., “I am quite capable of controlling my own emotions” and “I always encourage myself to try my best”). This scale consists of 16 items rated on a 7-point Likert-type scale ranging from 1 (strongly disagree) to 7 (strongly agree). This instrument elicits a global self-report EI score, with higher scores indicating higher self-report EI levels. Therefore, we combined the subscales into a global self-report EI measure as in previous studies (22, 36). This version of WLEIS has been proven to have good validity and reliability in Spanish populations [e.g., (22)]. In this study, Cronbach's alpha for WLEIS was 0.91.

Psychological distress was assessed using the Spanish version of the short-form Depression Anxiety and Stress Scales [DASS-21; (37, 38)]. This self-report instrument assesses psychological symptoms in the past week through a Likert-type scale (e.g., “I couldn't seem to experience any positive feelings at all” and “I felt I was close to panic”). Each item is rated on a 4-point scale, with “0 = did not apply to me at all” to “3 = applied to me very much, or most of the time.” We combined the subscales into a global psychological distress measure as in previous studies (39, 40). Hence, scores on the three subscales were summed, with scores coded so that higher scores showed higher psychological distress. The Spanish version of the DASS-21 has shown satisfactory psychometric properties in previous studies with community samples (34) and college students (38). Cronbach's alpha was 0.92.

Suicidal thoughts and behaviors were assessed with the Suicidal Behaviors Questionnaire-Revised [SBQ-R; (41)]. The SBQ-R consists of four items that assess different dimensions of suicidality: lifetime suicidal ideation and attempts (e.g., “Have you ever thought about or attempted to kill yourself?”), frequency of suicidal ideation in the past year (e.g., “How often have you thought about killing yourself in the past year?”), communication of suicidal behavior (e.g., “Have you ever told someone that you were going to commit suicide or that you might do it?”), and self-reported likelihood of future suicidal behavior (e.g., “How likely is it that you will attempt suicide someday?”). SBQ-R items are scored on a Likert-scale ranging from 0 or 1 (*never*) to 5 (*very often*) or 6 (*very likely*) so that higher scores indicate greater suicidal behavior. Items scores were summed to obtain a total score. For this study, the SBQ-R was professionally translated from English into Spanish using the back-translation method. The Spanish version of SBQ-R has shown adequate reliability in prior research (22). Cronbach's alpha was 0.79.

### Statistical analyses

SPSS 22.0 was used to analyze the data. First, we conducted Pearson correlation analyses to test whether self-report EI was associated with the proposed mediator (psychological distress) and outcome variable (suicide risk) in the hypothesized directions. Following Cohen (42), the correlation coefficients of 0.10, 0.30, and 0.50 represent small, medium and large effect size, respectively (42). Second, mediation analysis was conducted using the procedures recommended by Hayes (43) with total score of self-report EI as the independent variable (IV) and suicide risk as the dependent variable (DV). Psychological distress was tested as the mediator variable (MV) (43). In order to rule out the possibility that associations between self-report EI and suicide risk could be confounded by socio-demographic factors, age and gender were included as covariates.

Bootstrapping with 5,000 resamples was used in order to obtain parameter estimates for both total effect model and indirect effect model. In addition, we used the 95% bias-corrected confidence interval. If the interval does not contain a zero, then the indirect effect is considered statistically significant (*p* < 0.05). Mediation analysis was conducted with the Hayes macro PROCESS (43).

### Results

Descriptive statistics and Pearson correlations are reported in Table 1. All of the measures were significantly associated. As predicted, self-report EI scores showed significant negative correlation with psychological distress (*r* = −0.42; *p* < 0.01). Moreover, self-report EI was found to be correlated with suicide risk in the expected direction (*r* = −0.32; *p* < 0.01). Finally, psychological distress and suicide risk were positively related (*r* = 0.33; *p* < 0.01). According to Cohen's standard (42), the effect sizes of the correlations between self-report EI, psychological distress and suicide risk were medium.

**Table 1:** Descriptive statistics and bivariate correlations among the study variables in Study 1.

	***M***	***SD***	**Range**	**Min**	**Max**	**α**	**1**	**2**	**3**
1. Emotional intelligence	5.11	0.92	5.44	1.56	7.00	0.91	–		
2. Psychological distress	11.44	8.10	36.00	0.00	36.00	0.92	−0.42^**^	–	
3. Suicide risk	4.39	2.54	16.00	3.00	19.00	0.79	−0.32^**^	0.33^**^	–

N = 438.

** *p < 0.01*.

The results of the mediation analysis are summarized in Table 2. First, after controlling for age and gender, self-report EI was found to be significantly and negatively related to suicide risk (*B* = −0.90, *S.E*. = 0.13, *t* = −7.18, *p* < 0.001). Psychological distress was found to be significantly and positively related to suicide risk (*B* = 0.08; *S.E*. = 0.02; *t* = 5.08; *p* < 0.001). Second, inclusion of psychological distress reduced the association between self-report EI and suicide risk but this relationship remained significant (*B* = −0.62; *S.E*. = 0.13; *t* = −4.59; *p* < 0.001). Lastly, results of bootstrapping showed that psychological distress was a significant mediator of the relationship between self-report EI and suicide risk [estimate = −0.28; *S.E*. = 0.08, 95% CI = (−0.46, −0.15)].

**Table 2:** Indirect effects of Emotional Intelligence (EI) on suicide risk through psychological distress controlling for age and gender.

	**Total effect model**				**Indirect effect model**			
**Path**	***B***	***SE***	***t***		***B***	***SE***	***t***	**BCa 95% CI**
Age^a^	0.01	0.01	1.24		0.02	0.01	1.65	
Gender^a^	0.11	0.24	0.48		0.19	0.23	0.82	
EI – distress *(a*)	−3.66	0.38	−9.56^***^					
distress – suicide risk (*b)*	0.08	0.02	5.08^***^					
EI – suicide risk (*c*)	−0.90	0.13	−7.18^***^					
EI – suicide risk (*c′*)	−0.62	0.13	−4.59^***^					
EI - distress – suicide risk (*ab*)					−0.28	0.08		[−0.46, −0.15]
*R*^2^	0.11				0.16			
F (*df)*	17.61^***^	(3, 434)			20.42^***^	(4, 433)		

EI, Emotional Intelligence; distress, psychological distress symptoms. N = 438. a, b, c, and c′ represent unstandardized regression coefficients: a, direct association between Emotional Intelligence and suicidal behavior; b, direct association between psychological distress and suicidal behavior; c, total effect between Emotional Intelligence and suicidal behavior (not accounting for psychological distress); c′, direct effect between Emotional Intelligence and suicidal behavior (accounting for psychological distress); ab, indirect effect between Emotional Intelligence and suicidal behavior operating through psychological distress. Full mediation, c is reduced by ab to a non-significant c′; partial mediation, c is reduced by ab, but c′ remains significant; indirect only, ab, but no c and no c' initially. BCa 95% CI, bias corrected and accelerated 95% confidence interval; 5,000 bootstrap samples.

a Age and sex were covaried.

*** *p < 0.001*.

## Study 2: materials and methods

### Participants and procedure

Participants in this prospective study were 330 undergraduate students from the University of Malaga (264 females; 80%) with a mean age of 22.22 years and ranging from 18 to 61 years (*SD* = 5.53). The marital status of the participants was: 93% single, 1.2% married, 3.9% separated/divorced and 1.5% coupled. One subject did not indicate his marital status.

Students were asked if they were willing to participate in research on “associations between emotions and well-being.” Data were collected with the help of a team of research assistants. All participants completed the T1 survey and 310 participants (250 female; 80%, mean age = 22.11, *SD* = 5.46) completed the T2 survey 2 months later. Students were fully informed about the voluntary and anonymous basis of participation. It was made clear to them that their participation was voluntary and that all data would remain confidential. In this sense, participants could not be personally identified. In addition, it was made clear that they could stop participating in case they got distressed filling in the questionnaire. Participants completed the surveys as a group and received course credits for their participation in the 2-month prospective study. They were fully aware that by completing the questionnaires they were in fact providing informed consent to use this data in our research. In sum, common inclusion criteria were being aged above 18 years old, being enrolled in an industrial and organizational psychology course at the time of this survey and willingness to participate in the study. The surveys were administered in paper-and-pencil format with writing instructions and included sociodemographic factors (e.g., age, gender and marital status) together with scales measuring our main study variables.

### Measures

Self-report EI was evaluated using the Spanish version of the WLEIS (see description in Study 1). Cronbach's alpha was excellent in this study: α = 0.87 in Time 1 (T1) and α = 0.88 in Time 2 (T2). We administered the Spanish version of the DASS-21 to assess psychological distress (see description in Study 1). In this study, internal reliability was excellent (α = 0.91 in T1 and T2). Suicide risk was assessed with the Suicidal Behaviors Questionnaire-Revised (SBQ-R; see description in Study 1). In this study, Cronbach's alpha was 0.77 (in T1 and T2).

### Statistical analyses

First, we conducted Pearson correlation analyses to test the associations between self-report EI, psychological distress and suicide risk. We followed Cohen's (42) standard for estimating the correlation coefficient effect size (42). Second, we conducted a *t*-test on the outcome variable (suicide risk) assessed both at T1 and T2 in order to examine whether there were significant differences over time. In the case of significant differences in suicide risk from T1 to T2, this variable would be included as a control variable. Similarly to Study 1, mediation analysis was conducted using PROCESS (43), with a 5,000 bootstrapping sample and a 95% confidence interval to judge the statistical significance of mediation (43). SPSS 22.0 was used to analyze the data.

### Results

Table 3 reports descriptive statistics and correlations among our study variables at T1 and T2. As is shown, self-report EI was negatively and significantly associated with psychological distress in both T1 (*r* = −0.35; *p* < 0.01) and T2 (*r* = −0.27; *p* < 0.01). Similarly, self-report EI was significantly and negatively related to suicide risk in both T1 (*r* = −0.19; *p* < 0.01) and T2 (*r* = −0.21; *p* < 0.01). Finally, psychological distress and suicide risk were significantly and positively related in both T1 (*r* = 0.41; *p* < 0.01) and T2 (*r* = 0.29; *p* < 0.01). Following Cohen's standard (42), the effect sizes of the correlations self-report EI-psychological distress and psychological distress-suicide risk were medium, whereas the other correlations showed small effect sizes. In *post-hoc* analyses using the Fisher r-to-z transformation, we examined the correlations between self-report EI and suicide risk in both Study 1 and Study 2 (T1). Results showed that the community sample reported significantly higher associations than the college student sample (*z* = −1.90; *p* < 0.05).

**Table 3:** Descriptive statistics and bivariate correlations among the study variables at Time 1 and Time 2 in Study 2.

	***M***	***SD***	**Range**	**Min**	**Max**	**α**	**1**	**2**	**3**
**TIME 1**									
1. Emotional intelligence	5.30	0.73	4.06	2.69	6.75	0.87	−		
2. Psychological distress	9.43	6.87	36.00	0.00	36.00	0.91	−0.35^**^	−	
3. Suicide risk	5.05	2.83	16.00	3.00	19.00	0.77	−0.19^**^	0.41^**^	–
**TIME 2**									
1. Emotional intelligence	5.31	0.73	3.88	3.06	6.94	0.88	−		
2. Psychological distress	10.26	7.41	36.00	0.00	36.00	0.91	−0.27^**^	−	
3. Suicide risk	4.97	2.69	13.00	3.00	16.00	0.77	−0.21^**^	0.29^**^	–

M, Mean; SD, Standard Deviation. T1 N = 330. T2 N = 311.

** *p < 0.01*.

Paired samples *t*-tests found no significant differences in suicide risk between T1 and T2 [*t*_(310)_ = 0.67; *p* = 0.51], and so T1 suicide risk was not included as a control variable in the main analyses, in favor of the most parsimonious model. Table 4 reports the results of the mediation analysis. First, we found that sociodemographic factors, age and gender were significantly related to T2 suicide risk. After controlling for age and gender, self-report EI was found to be significantly and negatively related to T2 suicide risk (*B* = −0.85; *S.E*. = 0.21; *t* = −4.09; *p* < 0.001). Likewise, psychological distress was found to be significantly and positively related to suicide risk (*B* = 0.15; *S.E*. = 0.02; *t* = 7.09; *p* < 0.001). After psychological distress was included in the model, the association between self-report EI and suicide risk decreased and it did change the significance (*B* = −0.36; *S.E*. = 0.21; *t* = −1.77; *p* < 0.001). In particular, the direct effect of self-report EI on T2 suicide risk was no longer significant after accounting for the variance predicted by psychological distress. Results of bootstrapping revealed that psychological distress totally mediated the relationship between self-report EI and T2 suicide risk (estimate = −0.49; *S.E*. = 0.14, 95% *CI* = [−0.82, −0.26]). In sum, self-report EI showed a negative effect on psychological distress, which in turn was linked to decreased suicide risk 2 months later.

**Table 4:** Examination of the indirect effect of EI on Time 2 suicide risk through psychological distress.

	**Total effect model**				**Indirect effect model**			
**Path**	***B***	***SE***	***t***		***B***	***SE***	***t***	**BCa 95% CI**
Age^a^	0.05	0.03	1.86		0.06	0.03	2.17^*^	
Gender^a^	−0.89	0.37	−2.37^*^		−0.79	0.35	−2.27^*^	
EI – suicide risk (*c′*)	−0.36	0.21	−1.77					
EI – distress *(a*)	−3.20	0.51	−6.25^***^					
Distress– suicide risk (*b)*	0.15	0.02	7.09^***^					
EI – suicide risk (*c*)	−0.85	0.21	−4.09^***^					
EI - distress – suicide risk (*ab*)					−0.49	0.14		[−0.82, −0.26]
*R*^2^	0.07				0.20			
F (*df)*	7.83^***^(3, 307)				19.37^***^(4, 306)			

EI, Emotional Intelligence; distress, psychological distress symptoms. N = 330 (T1) and 311 (T2). a, b, c, and c′ represent unstandardized regression coefficients: a, direct association between Emotional Intelligence and suicidal behavior; b, direct association between psychological distress and suicidal behavior; c, total effect between Emotional Intelligence and suicidal behavior (not accounting for psychological distress); c′, direct effect between Emotional Intelligence and suicidal behavior (accounting for psychological distress); ab, indirect effect between Emotional Intelligence and suicidal behavior operating through psychological distress. Total mediation, c is reduced by ab to a non-significant c′; partial mediation, c is reduced by ab, but c′ remains significant; indirect only, ab, but no c and no c′ initially. BCa 95% CI, bias corrected and accelerated 95% confidence interval; 5,000 bootstrap samples.

a Age and sex were covaried.

^**^p < 0.01;

*** *p < 0.001*.

## Discussion

The proposed research aimed to examine a mediator model involving self-report EI, psychological distress and suicide risk adopting both cross-sectional and prospective designs in two independent samples. As expected, our results showed that self-report EI was negatively related to suicidal thoughts and behaviors in both community (22) and college student samples [e.g., (20)]. Similarly, the correlations between self-report EI and psychological distress were in line with those shown by Martins et al. (3) and more recent studies on EI and health-related indicators (17, 44). In line with prior research, our results show that self-report EI facilitates positive outcomes for individuals, thereby constituting a valuable resource in preventing suicide.

Our findings in study 1 suggest that self-report EI may explain suicide risk both directly and indirectly through its influence on psychological distress. Therefore, individuals who perceive themselves more skilled in perceiving, understanding and managing their own emotions and the emotions of others seem to show decreased suicidal thoughts and behaviors via reduced psychological distress (30, 45). Study 2 helped us verifying these findings providing prospective evidence on the protective role of self-report EI on suicide risk through maintaining lower emotional distress (27).

Based upon prior research, self-report ability EI seems to be related to individuals' beliefs in their emotional skills to cope with threating events (46). Relatedly, the influence of self-report EI on lower suicide risk might occur through encouraging development of adaptive strategies that decrease the individuals' vulnerability toward negative mood states associated with the likelihood of suicidal thoughts and behaviors (22, 27). For instance, EI appears to be linked to the use of certain coping strategies such as rumination, social support seeking or emotional disclosure (47). In the same vein, a recent meta-analysis on EI and emotion regulation strategies has provided empirical evidences on the fact that higher EI individuals tend to regulate their emotions and display less emotional reactivity in response to negative emotion-eliciting events (29). In sum, individuals' beliefs in their emotional skills to deal with demanding events might reduce emotional distress symptoms that might, in turn, be key factors in determining the frequency and intensity of future suicidal thoughts and behaviors (17, 22).

## Limitations and future research

Several limitations of this work should be considered because of its implications for future research and practice. First, a limitation of the present work may be constituted by the common method variance derived from the use of self-report measures (3). Nonetheless, the construct validity of our study variables encourage us to find this question less problematic (48). Although most of the studies in the field of EI and suicide have used self-report ability EI tests with adequate psychometric properties, future studies are advised to examine jointly both performance-based and self-report instruments of EI (2, 49). In addition, semi-structured interviews or observers' ratings of EI are advised to complement the main approaches to assess ability EI. Relatedly, although performance-based ability EI has shown incremental validity in explaining suicidal ideation above the variance accounted for by personality traits (28), further research is needed to consider the potential influence of dispositional factors on suicide risk [e.g., (50)].

Second, we used a self-reported measure of psychological distress rather than instruments assessing psychopathological factors. Although further research should include additional measurement methodologies, such as expert judgments or clinical diagnosis, assessment of psychological distress symptoms undoubtedly constitutes a promising line in psychiatric research (51). Although we assessed marital status as well as previous studies on EI and suicide risk did [e.g., (18, 22)], future studies are advised to examine other important sociodemographic factors such as educational level, which might function differently depending on the levels of educational attainment. Finally, although gender was controlled in our analyses, prior research has shown differences between males and females in rates of psychological symptoms [e.g., (52)], along with the prediction of suicidality (53). Therefore, gender specific moderated mediation models should be considered in future studies (54).

One of the contributions of our work is that self-report EI was found to both cross-sectionally and prospectively predict suicide risk through its influence on psychological distress. In this sense, it is noteworthy that psychological distress partially mediated this relationship in Study 1, whereas it fully mediated the association between self-report EI and T2 suicide risk in Study 2. One plausible explanation for this difference might be due to the nature of the sample. It is tentative to assume that suicide risk in the community sample may be more externally determined and depend on a higher variety of contextual and sociodemographic factors that are traditionally related to psychological distress [e.g., (14, 15, 32)]. Conversely, psychological distress symptoms might be more determinant of suicidal thoughts and behaviors in a more homogenous sample constituted by college students [e.g., (16, 55)]. Undoubtedly, future studies comparing relatively large samples are advised to replicate these findings. In addition, further research adopting longitudinal and experimental designs is needed to broaden the current understanding of the protective role of EI in suicidal thoughts and behaviors. Although sampling bias could be a potential limitation of the snowball sampling technique used in study 1 (56), the instructions on the questionnaire were brief and precise aiming at avoiding these biases to a greater extent.

Taken as a whole, our findings add support to the assumption that EI might help alleviate emotional distress, thereby decreasing the likelihood of suicidal thoughts and behaviors. These results highlight the role of EI as a promising line of intervention in preventing psychological maladjustment and suicide thoughts and behaviors (57, 58). Besides, given the literature focusing on the crucial role of negative emotional states as precursors of lower physical and mental health [e.g., (3, 17)], these findings might be valuable when designing population-based interventions (12, 57). Accordingly, interventions that target both an alleviation of psychological distress and negative mood states (59) and an increase in emotional abilities (60) may offer the most promise in working with individuals experiencing higher suicide ideation (58). In sum, our results point out the potential value of using EI-based stress reduction interventions that specifically assess and that target deficits in affective mechanisms regarding mental health-related outcomes as a potential means for reducing suicide risk (61).

With respect to practical implications derived from our findings, interventions on EI might be useful in order to increase individuals' set of adaptive emotion regulation strategies (29). The development of emotional abilities might help to increase perceived social support that, in turn, is related to lower barriers regarding help seeking behaviors (5, 62). In this context, prevention programs including EI training would be relevant for individuals in obtaining support from available services [e.g., (55)]. Given the potential value of preventive intervention programmes aiming at increasing access to mental health services (12), this line of research merits serious attention. In addition to intervention programmes targeting classic precursors of suicide such as mood dysregulation (12, 63), EI training might help individuals breaking the cycle of increasingly negative and constricted negative thinking linked to risk of suicide (19, 63). Furthermore, complementary interventions through occupational or academic training programmes might increase positive emotional states and, hence, the development of physical, social and psychological resources (58, 60). In sum, our findings open the door to future practical implications with the aim of helping individuals build a system of resiliency to sources of academic and occupational stress that might lead to impaired health risk of suicide (58).

## Conclusion

The present work provided evidence on the mediating role of psychological distress in the association between self-report EI and suicide risk across samples (community sample and college students) using both cross-sectional and prospective designs. To the best of our knowledge, no study have been conducted to test the prospective effects of EI on suicide risk nor the explanatory mechanism by which EI may prevent suicidal thoughts and behaviors.

These findings provide preliminary evidence for the crucial role of self-report emotional abilities in reducing suicidal thoughts and behaviors via reduced psychological distress. Nonetheless, much research is needed to examine the influence of mediating and moderating factors involved in this complex association. Given the alarming prevalence of suicide as a complex public health concern, this line of research linking emotional processing with health-related outcomes requires further attention.

## Ethics statement

Since the Spanish law does not impose the requirements of ethics approval nor written informed consent in case of self-report and anonymous research carried out with healthy subjects, ethics approval was not needed in the present research. Nonetheless, participants were fully informed about the voluntary and anonymous basis of participation. In addition, they were fully aware that by completing the questionnaires they were in fact providing informed consent to use this data in the present research.

## Author contributions

SM-L, NE, and LR are responsible for study conception, design and implementation, data analyses and interpretation. All the authors worked on the first draft of the work, reviewed, and approved the final manuscript.

### Conflict of interest statement

The authors declare that the research was conducted in the absence of any commercial or financial relationships that could be construed as a potential conflict of interest.
